# Influence of Major Public Health Emergencies on Family Relationship and Humanistic Geographical Characteristics of China

**DOI:** 10.3390/ijerph18083879

**Published:** 2021-04-07

**Authors:** Xiaojia Guo, Jingzhong Li, Yexin Gao, Fang Su, Bing Xue

**Affiliations:** 1 Department of Human Geography, College of Geographical Science, Shanxi Normal University, Linfen 041000, China; guoxj@sxnu.edu.cn (X.G.); gaoyexin@stu.sxnu.edu.cn (Y.G.); 2 Departmentof Resource Utilization and Environmental Rehabilitation, Institute of Geographic Sciences and Natural Resources Research, Chinese Academy of Sciences, Beijing 010000, China; 3 Department of Geographic Information Science, College of Urban Planning and Architecture, Xuchang University, Xuchang 461000, China; 4 Department of Technical Economy and Management of Light Industry, School of Economics and Management, Shanxi University of Science & Technology, Xi’an 710021, China; sufang@sust.edu.cn; 5 Department of Industrial Ecology and Sustainability, Institute of Applied Ecology, Chinese Academy of Sciences, Shenyang 110016, China; xuebing@iae.ac.cn

**Keywords:** major public health emergencies, family relationships, humanistic characteristics, spatial distribution patterns, influence factors

## Abstract

Harmonious and stable family relations are undoubtedly an important component of victory in terms of epidemic prevention. Take the COVID-2019 (2019 new crown pneumonia epidemic) as the major public events background; 24,188 national samples were obtained based on a network survey. We selected gender, education level, occupation type, family scale, neighborhood relationship and psychological state as independent variables, and adopted multiple logistic models to assess the impact of major public events on family relationships and the characteristics of humanistic–regional attributes. The findings are as follows: (1) During the epidemic period, major public health emergencies effectively promoted the national residents’ family relationships. (2) The family relationships of national residents presented a high level in central China and a low level in the border areas of China, which is consistent with the spread of COVID-2019 in January and February. (3) Family relationship level averages between 2.201~2.507 among different groups when divided by occupation, age and education. The family relationship has improved, but the change is not drastic and the gap between various groups is not significant, so there is essentially no difference. (4) The impact of major public health emergencies on all families is nearly sudden and instant, so that family relationship changes are often also abrupt. (5) Educational level, family size and gender have a positive effect on the change in family relations, but this effect is weakened as family education level increases; while the anxiety of the interviewees and the neighborhood had a negative effect on the change in family relationship, this indicates that the better the neighborhood relations are, the more harmonious a family relationship is. The above research can provide an important scientific support and decision-making basis for the government to carry out community prevention work, respond to major public health emergencies and construct a family support social policy system in the future.

## 1. Introduction

Family relationship and community are the basis of social bonds that form a stability spanning time and space [1] and gradually become important research topics of the humanities, social geography and other disciplines. Active communication among family members is conducive to avoiding dialogue barriers or understanding deviations that may be caused by generational gaps [2], which is conducive to personal health and is useful to improve learning, work and social adaptability [3]. In recent years, different scholars have discussed family relations from the perspectives of sociology, psychology and management, such as the intergenerational family relations of the elderly [4,5,6], parent–child relations [7,8,9] and the impact of the Internet and new media on traditional family relations [2,10,11]. From the perspective of human and social geography, family daily life is constructed under the political and economic constraints present at a given specific time and place, and its significance to individual growth and social progress changes over time [1]. It usually transfers or changes in the context of different events (such as major public health emergencies) [12].

Looking back at human history, infectious disease diseases, natural disasters and other emergencies have always accompanied human society [13], resulting in a large number of casualties and heavy economic losses, threats to both families and society and aggravations in the instability and vulnerability of national and regional development. President Xi Jinping of China clearly pointed out that “no matter how to change, no matter how economic and social development, for a society, family daily life cannot be replaced, the social function of family cannot be replaced, family plays an irreplaceable role in civilization” [14], and what’s more, he stressed that “families are in good status, the country and national society are stable” [15]. Family harmony contributes to social stability and national security, especially for the special groups, such as the elderly [4,5,6], teenage children [8,9], high-risk medical groups [16,17], patients [18,19] and vulnerable groups [20]. Harmonious and stable family relations can effectively alleviate nervous emotions caused by public health emergencies and can provide psychological support for families to enhance their ability to tolerate, cope with and escape from stressful situations. Since ancient times, the Chinese people have attached great importance to family affection and have regarded a loving family relationship as the cultural consciousness and spiritual wealth of the Chinese nation [15]. Therefore, in the context of major public health emergencies, it is worth exploring whether it is beneficial to the improvement of family relations for families to spend a long time together, the evaluation of which requires a huge social experiment.

The 2019 new crown pneumonia epidemic (COVID-19) has brought a comprehensive impact on people’s social and economic lives. Accordingly, many governments have closed off their cities in order to isolate people and goods to halt the spread of the disease around the world, which has had different impacts on family relationships and has caused great changes in people’s daily lives [21]. The existing studies have shown that the level of public security in public emergencies is usually an intuitive dimension to measure the degree of national modernization [21,22,23]. Meanwhile, China is in a critical period of social transformation and modernization, and the high frequency of major public health emergencies poses a new challenge to the national governance system and its capacity [22]. Hence, it is of great significance to systematically reveal the changes to family relations and their regional characteristics in the context of major emergency events, which will be conductive to elaborate and promote sustainable regional governance measures and their long-term mechanisms.

## 2. Materials and Methods

### 2.1. Questionnaire Design

In order to analyze the changing trend of household relations in the course of the COVID-19 outbreak, this questionnaire mainly consists of two parts. The first part is intended to collect the basic information of the interviewee, mainly including age, gender, the province they are living in during the epidemic, the address of residence during the epidemic, education level, occupation type, family size and psychological state. The second part includes four questions, such as the interaction time, communication content, communication depth and neighborhood relationship of the interviewees, shown as follows:

Question 1. The interaction time with your family (including remote communication, face-to-face communication with family and with family activities such as playing games, etc.) compared to before the epidemic. Answer: (A. increased significantly); (B. slightly increased); (C. no change); (D. slightly decreased); (E. decreased significantly).
Question 2. The main content of communication with family members living together. Answer: (A. data change about the epidemic); (B. discussion on social topics caused by the epidemic); (C. family life experience or future planning); (D. social topics or ideas); (E. entertainment, sports or international news events); (F. family interests and hobbies); (G. chatting).
Question 3. The communication with your family members is shallow or deep? Answer: (A. shallow communication); (B. deep communication); (C. cannot be judged).
Question 4. The relationship with neighbors compared to that before the epidemic. Answer: (A. is significantly better); (B. is somewhat better); (C. is basically unchanged); (D. is somewhat worse); (E. is significantly worse).

### 2.2. Questionnaire Collection

Since the end of January 2020, in order to stop the spread of COVID-19 and curb nationwide spread, Wuhan has been declared on “lockdown” since 10 a.m. on 23 January 2020. Subsequently, all parts of China successively launched their first-level responses to public health emergencies and adopted the most comprehensive, strict and thorough prevention and control measures [24]. For example, urban public transport, subway, ferry and long-distance passenger transport services were suspended, and restaurants, bars, factories, schools and other densely populated places were temporarily closed. People have had to comply with the measures: be isolated and stay at home. Students could not go to school, and workers could not go to the factory. Once individuals went out, they had to wear masks to keep a safe distance of 1 m. Fortunately, one month later (on February 24), the State Council Joint Prevention and Control Mechanism news conference announced that the number of newly diagnosed cases in mainland China had been less than 1000 cases for five consecutive days [25]. Additionally, the number of newly diagnosed cases in most provinces (except for Hubei) had been in a single digit trend since February 27. The number of existing diagnosed cases showed a downward trend. The number of cured and discharged cases in all provinces was greater than or equal to the number of newly diagnosed cases, and the epidemic had been effectively controlled.

Although the epidemic situation is different in various provinces, all residents face the same policy that has kept them in quarantine at home and utilizing the same sources of information from the Internet, so we can’t carry out offline questionnaires and can only carry out online questionnaires. Therefore, from February 21 to 28, 2020, a total of 24,215 samples were collected based on snowball sampling, among which 24,188 were valid, with an effective rate of 99.89%. Through data cleaning, data entry and other preprocessing, 27 incomplete surveys, all coming from the same source, were filtered out. SPSS 17.0 software (IBM, Armonk, NY, USA) was used to preprocess individual missing data in the questionnaire, and the original data are supplemented by the mean substitution method. The survey covered 2463 county-level administrative units (municipal districts or county-level cities) in 31 provincial-level administrative regions (excluding Hong Kong, Macao and Taiwan). Among them, there were 699 samples from Hubei Province, 951 samples from Shandong Province, 782 samples from Beijing City, 786 samples from Guangdong Province, 1310 samples from Sichuan Province, 440 samples from Heilongjiang Province, 456 samples from Zhejiang Province, 685 samples from Hunan Province, 1053 samples from Chongqing City, 447 samples from Guangxi Province, 1801 samples from Gansu Province, 703 samples from Guizhou Province, 415 samples from Shanghai City, 1876 samples from Jiangsu Province, 604 samples from Jiangxi Province, 1178 samples from Liaoning Province, 1425 samples from Shaanxi Province, 1067 samples from Shanxi Province, 343 samples from Inner Mongolia, 610 samples from Henan Province, 1571 samples from Anhui Province, 262 samples from Tianjin City, 699 samples from Hebei Province, 543 samples from Hainan Province, 437 samples from Ningxia, 888 samples from Yunnan Province, 735 samples from Jilin Province, 482 samples from Xinjiang, 228 samples from Fujian Province, 628 samples from Fujian Province, 668 samples from Qinghai Province and 44 samples from Tibet.

In the valid questionnaire, there were 14,493 female respondents (accounting for 59.9% of the total), and the group aged 20–49 accounted for 76.2% of the total. The nuclear family with 3–4 members accounted for as much as 60% of the total, which is consistent with China’s basic national policy of family planning that has been in place for many years, and this is consistent the research results of Feng, Wang and others [26,27,28]. As a result, family size is getting smaller and smaller, with the average family size dropping from 4.41 in 1982 to 3.17 in 2006 [29]. Additionally, 21,819 individuals with college degrees accounted for 90.2% of the respondents. In terms of type of occupation, college students accounted for the highest proportion of 53.1% of the respondents, followed by employees of enterprises and public institutions, which accounted for 27.7% of the respondents, which is consistent with the education levels of the interviewees.

### 2.3. Research Method

#### 2.3.1. Quantitative Assignment Method

The change in family relationship characterization is up to the self-comparison of interviewees (before the epidemic and after the outbreak), and family interaction level (including remote communication, face-to-face communication with family and family activities such as playing games, etc.) is divided into “significant increase, slightly increased, no change, slightly decreased and a significant reduction” five levels. Similarly, the respondents ‘relationship with their neighbors are divided into five levels: significantly better, slightly good, no change, some variation, and remarkable. For the two items, a 5-level Likert scale was used to assign one to five points from high to low. The higher the score, the weaker the neighborhood and family relationship of the interviewees, probably because lack of communication during the quarantine in the home due to the outbreak of the epidemic.

#### 2.3.2. Spatial Association Analysis

This study used ArcGIS software (Esri, Redlands, CA, USA) to calculate the global Moran coefficient to study the spatial agglomeration of family relationship closeness. Spatial autocorrelation can be understood as similar variable values in neighbor regions with similar positions. If the high value and the high value gather together, and the low value and the low value gather together, this is a “positive spatial autocorrelation“. Conversely, if the high value is adjacent to the low value, this is a “negative spatial autocorrelation”. Additionally, if the high value and low value are completely random in location, there is no spatial autocorrelation. Based on the complexity of spatial autocorrelation, the global Moran index (Formula (1)) and the local Moran index (Formula (2)) are used to measure the global spatial correlation between adjacent regions and the local spatial correlation between regions and peripheral regions.

(1) $$I=\frac{\sum_{i=1}^{n}\sum_{j=1}^{n}w_{ij}\left(x_{i}-\bar{x}\right)\left(x_{j}-\bar{x}\right)}{S^{2}\sum_{i=1}^{n}\sum_{j=1}^{n}w_{ij}}$$

(2) $$I_{i}=\frac{\left(x_{i}-\bar{x}\right)\sum_{j=1}^{n}\left(x_{j}-\bar{x}\right)}{S^{2}}$$

(3) $$S^{2}=\frac{\sum_{i=1}^{n}{\left(x_{i}-\bar{x}\right)}^{2}}{n}$$

where, *n* is the number of the provinces, $S^{2}$ is sample variance, $w_{ij}$ is the (i,j) element of a spatial weight matrix. The value of Moran’s I ranges from (–1,1). When its value is positive, this indicates that there is a spatial positive correlation, and if its value is negative, this indicates a spatial negative correlation. When the value approaches 0, there is no significant spatial correlation. The larger the absolute value, the stronger the spatial correlation. Based on the geographical weight matrix, it is closely related to the household relationship where possesses similar spatial characteristics.

#### 2.3.3. Multiple Logistic Regression Models

In order to further analyze the relationship between family relations and physical quality, cultural literacy, professional experience, social relations and psychological state during the epidemic, we selected gender, education level, occupation type, family scale, neighborhood relationship and psychological state as independent variables which passed the significance level test, we selected family relationship as a dependent variable and we adopted multiple logistic models for empirical analysis. The form is as follows:

(4) $$status_{i}=edu_{i}+gender_{i}+occupation_{i}+scale_{i}+mood_{i}+neighborhood_{i}+\varepsilon_{i}$$

where, subscript *i* represents the *i*th sample and *ε* is a random perturbation term. The dependent variable *status_i_* is a measure of household relations for the *i*th sample. The independent variables included the education level (*edu*), *gende*, occupation type (*occupation*), family size (*scale*), psychological state (*mood*), *neighborhood* and so on. The variables in the model are explained in Table 1.

## 3. Results

### 3.1. Overall Change Characteristics

**Figure 1:**
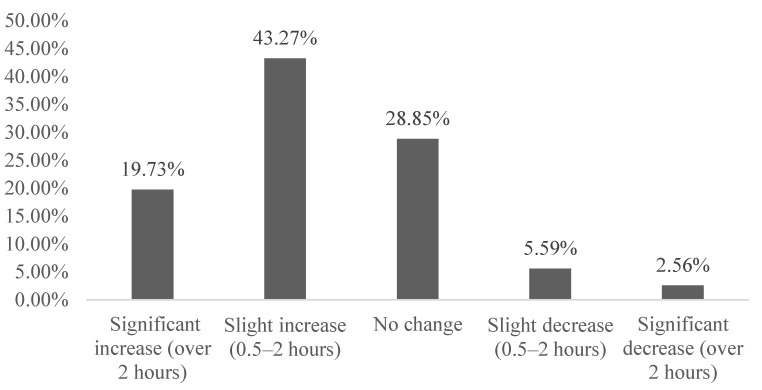
Changes in interaction between the interviewees and their family member under the epidemic.

The results of the investigation and evaluation showed that the mean value for household interaction was 2.28, the mode was 2, and the change in family relationship was positively skewed. In particular, 63% of respondents said they had more contact with their families than before the epidemic (such as in network communication, face-to-face family activities and playing games), while only 8.16% of respondents said they had less communication with their families. As a result of COVID-19, the topic of family members’ communication increased and the emotional communication deepened, which contributed to the improvement of family relations (see Figure 1). This result also consistents with individual case interviews. Interviewee A said, “the relationship between her parents and her has become more harmonious, because the epidemic period is the longest stay at home since become a college student, but sometimes stay longer will be boring”. However, respondent B said “the desire to go to work, since staying at home only expenses without income, living become difficult”. Correspondingly, 88.2% of the respondents said that the relationship with their neighbors remained basically unchanged, since because of the specificity of the epidemic spread and the characteristics of human transmission, the central and local government tried their best to control epidemic prevention, limiting planes and trains all over the country and suspending buses, subways and ferries alongside other public transport. Probably because residents quarantining in their own home, avoiding visiting relatives and friends and attending parties, obviously reducing neighborhood contact, thus basically resulting in no impact on neighborhood relations. However, 3.2% of respondents said that neighborhood relationships were worse than ever due to panic and material stress.

**Figure 2:**
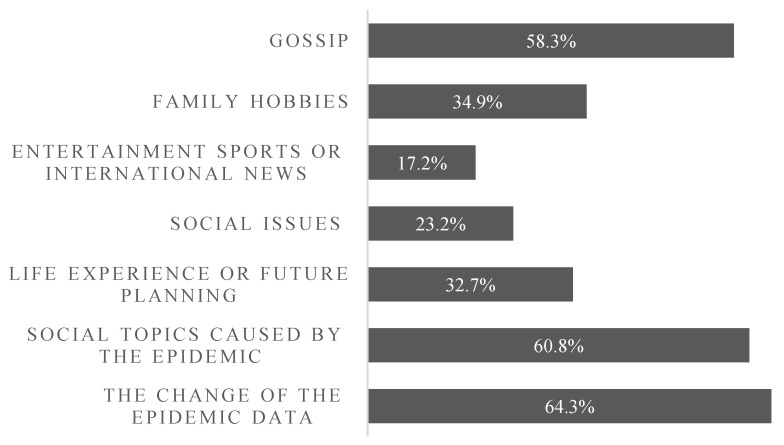
Key elements of communication between the interviewees and their family member.

In the closed or semiclosed home environment, family members are more inclined to talk about hot issues related to the epidemic situation. The proportion of the group whose main communication content is the change in epidemic data was measured up to 64.3% of the total, and the percentage of groups that discussed the social topics caused by the epidemic was as high as 60.8% of the total. In terms of being affected by the epidemic and the closed lifestyle, on the one hand, a large proportion of people who have been idle at home for a long time chat and kill time, which accounted for 58.3% of respondents; on the other hand, there has been an increase in emotional communication among families living together, with nearly a third of respondents sharing life experiences, future plans and hobbies with their family members. In terms of the degree of communication between family members, the largest proportion of respondents with superficial communication accounted for 42.6% of the total, while 29.1% of respondents had in-depth communication with their families to exchange ideas and 28.2% of respondents said that they could not judge the degree of communication. To the contrary, only a small proportion of respondents were concerned with entertainment or international news events, only accounting for 17.2% of the total (see Figure 2).

Man is the active factor of the relationship between humanity and the land, and it is also the core element affecting socio–economic geography [30]. This is especially true when entering the era of intelligent information and ecological civilization in the 21st century, wherein personal moral belief, mode of thinking and spiritual pursuit are the endogenous driving forces for familial harmony. According to the results of the survey, the mean value of family relationship interaction among different groups ranged from 2.201 to 2.507 (see Table 2). Compared with the threshold of “1–5” (corresponding to “significant increase—significant decrease”), the overall communication and interaction among family members generally increased, but the change in family relationship was not drastic and the gap among different groups was not significant, which essentially amounted to no difference. This shows that the impact of public health emergencies on all families is the same, regardless of the individual’s occupation, age, education level and family scale. The most important thing for the family relationship is the time spent together, and the companionship of the family members is fundamental to family harmony and stability. It is noteworthy that the impact of public health events is sudden and the time node is very strong, which is different from the findings of a long-term in-depth investigation and interview from the society or psychology perspective [31,32,33]. The changes in family relationships caused by this may also be abrupt, rather than the subtle influence of family members over multiple years.

### 3.2. Spatial Distribution Characteristics

**Figure 3:**
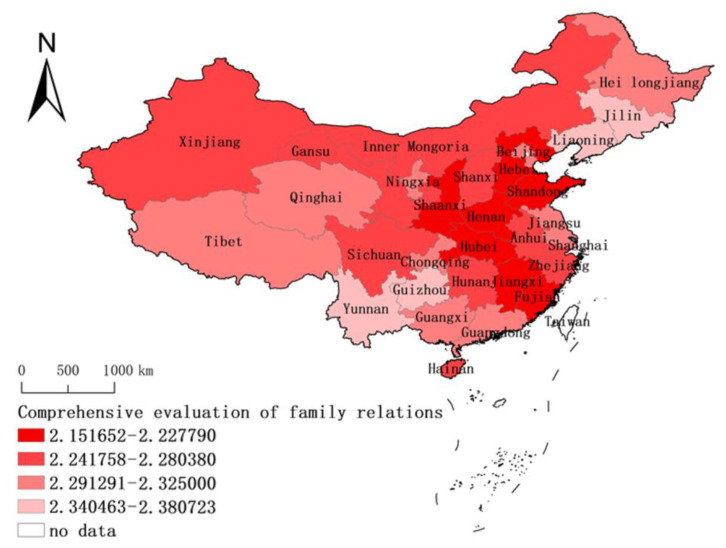
Spatial distribution characteristics of the comprehensive family relationship assessment.

Based on the calculation and analysis of the whole sample, the family relationship of 31 provinces in China was evaluated at the provincial level and its spatial distribution characteristics were described (see Figure 3). According to the comprehensive evaluation of family relations in 31 provinces in China, it was found that the family relations of residents in China are generally harmonious, with a high frequency of family communication and interactions, and the average score of each province is less than three, but there is still a deviation. Compare to other provinces, Henan residents have the closest family relationship, with an average of 2.152, indicating that the intensity and frequency of family activities (face-to-face and Internet emotional communication) increased significantly under the epidemic. On the contrary, the lowest mean value of the family relationship was found in the Yunnan province at 2.381 which less than three, indicating that family interaction had been improved under the epidemic, but the degree of ascension was the lowest compared with other provinces.

**Figure 4:**
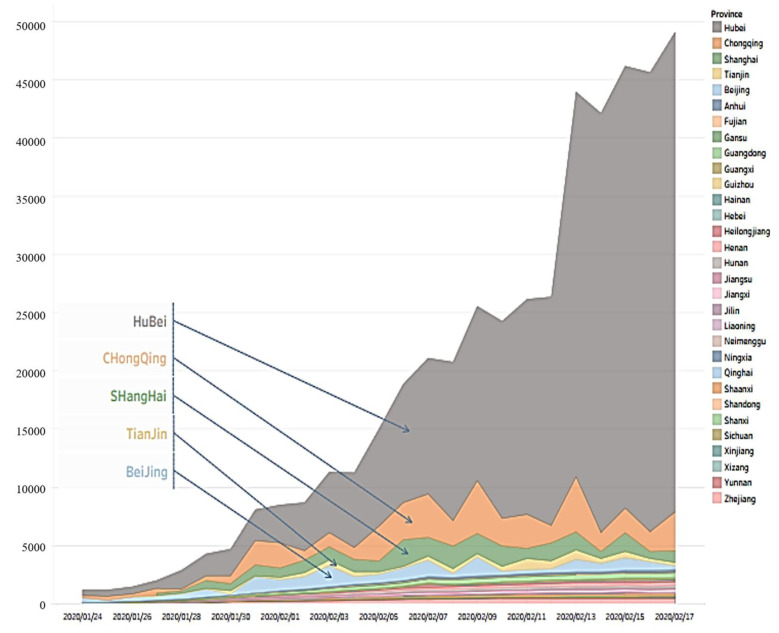
The changes in the number of new COVID-19 cases in various provinces of China from January 24 to February 17.

Based on the natural discontinuity method, the degree of family interaction and communication in 31 provinces of China was divided into four levels, with one to four representing the degree of family closeness (see Figure 3). Provinces at level one included Liaoning, Guizhou, Jilin and Yunnan. More than one-third of the provinces were at level two, including Guangdong, Ningxia, Heilongjiang, Qinghai, Guangxi, Beijing, Chongqing, Shanghai, Jiangsu, Tianjin and Xizang. Nearly one-third of the provinces were at level three, including Inner Mongolia, Hong Kong, Shanxi, Hainan, Zhejiang, Hunan, Sichuan, Xinjiang, Gansu and Anhui. Level four included Henan, Shandong, Hubei, Shaanxi, Jiangxi, Hebei and Fujian. Overall, the family relations of Chinese residents present a low–central and border–high spatial distribution pattern, which is consistent with the spread situation of COVID-19 in China during January and February as shown in Figure 4. In the wake of the epidemic, families offer each other significant emotional support, especially in the Hubei province, which is severely affected by the epidemic. Additionally, in its neighboring provinces of Shaanxi, Henan and Jiangxi, family relations have also been greatly improved. On the contrary, the epidemic situation in the provinces of Jilin, Liaoning, Yunan and Guizhou is relatively light compared to other provinces during January and February, and thus the local people face less psychological pressure than those in other regions and panic tension is low, and family communication has slightly increased.

**Figure 5:**
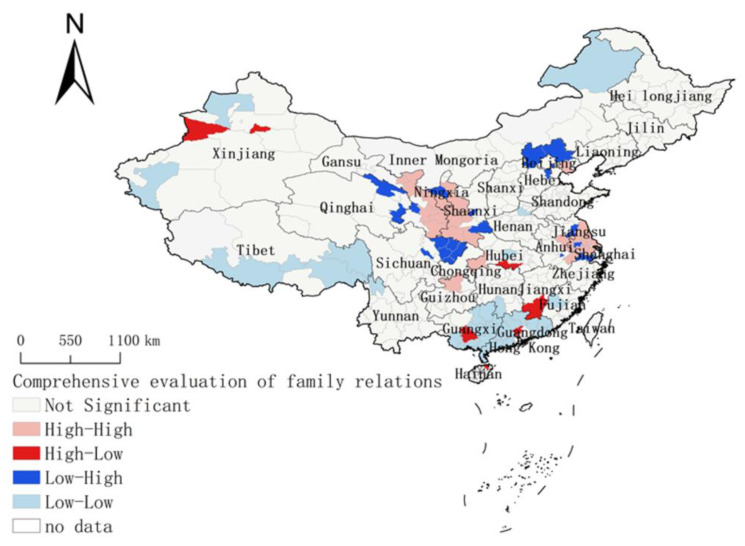
Lisa gathering of family relationship closeness.

The spatial clustering of family closeness was studied based on the geospatial correlation method (see Figure 5). According to the research results, the global Moran’s I index is 0.0485, the sample variance is 0.0002, the Z score is 3.4714 and the *p* value is 0.0005, indicating that the probability of the agglomeration of public family relations in geographical space is less than 1%, which mainly presents a random distribution pattern of a plaque mosaic. It is worth noting that the Shaanxi–Gansu–Ningsia area and the Jiangsu–Anhui area represent “high–high” agglomerations and the Guangxi–Guangdong area and the border area of Xinjiang–Tibet–Inner Mongolia represent “low–low” agglomerations; that is, the Guangdong and Guangxi areas and the ethnic border area are the agglomeration areas with lower family relations, and the Jiangsu–Anhui area and Shaanxi–Gansu–Ningsia border area are the agglomeration areas with higher family relations. The differentiation of “high–high” and “low–low” in terms of family relations indicates that there is an obvious dependence and heterogeneity in the local spatial distribution of public family relations in China during the COVID-19 epidemic period. There is a positive spatial spillover effect of family relations in the Shaanxi–Gansu–Ningsia areas and the Jiangsu–Anhui region, while a negative spatial spillover effect of family relations is present in the Guangdong–Guangxi area and the Xinjiang–Tibet–Inner Mongolia ethnic minorities area. At the same time, there is no spatial spillover effect between Hubei and its neighboring provinces, and there is no “high–high” agglomeration or “low–low” agglomeration of family relations among Hubei and its surrounding provinces, which indicates that COVID-19 has no significant proximity effect on residents’ family relationships on a provincial level.

**Figure 6:**
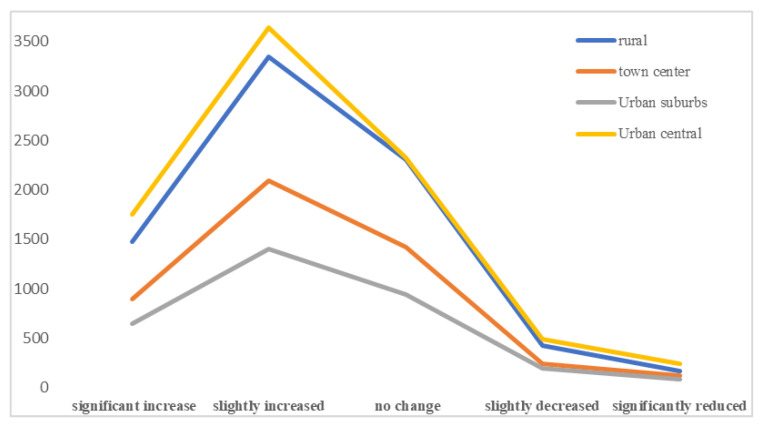
The interaction of family members in different settlements.

In terms of urban and rural settlements, due to the long-term urban–rural dual system in China and the uneven development policies of urban and rural areas, as well as the different natural landscapes and cultural backgrounds present, correspondingly different economic pattern and life philosophies have been formed [34], which may lead to cognitive biases and regional differences in family relations between urban and rural residents. Whether in urban or rural areas, the survey reflects that most of the respondents have improved their family relationships under the epidemic, while few family relationships have become worse, as shown in Figure 6.

### 3.3. Influencing Factors Analysis

The change in family relationship was selected as the dependent variable, and age, gender, education level, occupation type, family scale, neighborhood relations and psychological state were selected as explanatory variables to carry out influencing factor analysis. From Table 3, it is known that gender, education level and family scale have a positive influence on family relationship, and all of them are significant at a 95% level. Meanwhile, occupational type, neighborhood relations and psychological state during the epidemic had a negative impact on family relationships, with significance levels of 95%, 95% and 90%, respectively. However, age had a positive impact on family relationships, but failed to pass the significance test.

The likelihood ratio test showed that the significance level *p* of the chi-square value of the multinomial logistic model was 0.000, which indicates that the model fitted well and the simulation results were reliable. The following results can be obtained from Table 4:

(1) Except for students, the changes in family relationships caused by different occupational groups are different. Since the score of family interaction is a negative indicator, the higher the value, the less significant the family relationship. Therefore, the family interaction and contact of other groups besides students are significantly improved. In term of occupational types, “governmental employees”, “middle-level cadres” and “entrepreneurs” are all significant at a 1% confidence level. This is possibly because these groups are often the core of the family soul and economic pillar and are familiar with WeChat, Weibo, QQ and other telecommunication tools. These groups usually soak themselves in busy work and neglect family, and they have more time to accompany their family and more in-depth communication and interaction with other members during the epidemic period; this is basically consistent with the research results of Cheng, Chang and others [18,35]. The change in family relationship of the student group is positive and significant at a 1% confidence level, which indicates that the relationship between college students and their families appears sensitive and tense in the long-time closed environment. It may be due to full cover type home quarantine that students are trapped with families in the home, and it is easy to produce problems such as disagreement and misunderstanding, which is consistent with Zhao’s research conclusion [21].
(2) The influence of gender and psychological status on family relationship is significant at a 1% confidence level. Gender has a positive effect on the family relationship, indicating that gender differences between men and women show significant differences in family relations stimulated by public health emergencies. This is probably because the men and women play different role in society and social expectations on men and women are inconsistent. Because of women being more sensitive and sentimental than men, more prone to anxiety and depression than men and more likely to create conflict and tension with their families, which is basically consistent with the findings of Qin and Cheng [18,36]. Under the epidemic situation, the stress of the respondents has a negative impact on family relations. The lack of critical information and insufficient knowledge regarding the disease gave rise to the spread of public tension and panic, which ultimately led individuals to seek the comfort and companionship of their families. Zhao and Wei’s research has also shown that family work and recreational activities together can distract from panic, release pressure and alleviate the negative feelings of residents in dealing with the epidemic [20,21].
(3) Family scale and education level have a positive influence on the changes in family relations and has passed the significance level test. Coupled with the increase in family size, this effect gradually decreases, showing that the influence of nuclear families is higher than that of traditional families, which in turn shows that the probability of a worse family relationship with fewer family members is much higher than that of a family with a larger family size. This is perhaps because the conflicts and problems of small family tend to be magnified, while the contradictions and problems of large family can be adjusted and alleviated by other members.
(4) Education level has a positive influence on the change in family relationship and has passed a significant level of 1%. However, with the improvement of education level this influence weakened, indicating that the probability of a family relationship becoming worse for people with a low education level is much higher than that of people with a high education level. Due to the education level higher, the public has a more scientific understanding of the epidemic situation. Along with this, the highly educated groups generally have a higher knowledge reserve gleaned through the school biological health course and other courses, which enables them to deal with the epidemic situation more clearly and rationally and take effective prevention and control measures. In addition, they are also more likely to guide their families and conduct their own psychological construction. Relevant surveys also show that education level is an important factor affecting people’s cognitive levels [37,38,39].
(5) Neighborhood relationship has a negative effect on the change in family relationship and passed the significance test; that is, the more harmonious a neighborhood relationship is, the greater its influence is. This shows that the probability that the family relationship of a respondent becomes better with better neighborhood relationships is much higher than that of respondents with worse neighborhood relationships, indicating that social support has a positive effect on the improvement of family relationships, which is basically consistent with the research results of Jiang and Wen [40,41].

## 4. Discussion

Chinese President Xi Jinping has said, “We need to realize that only when every family is harmonious can the country and nation be thriving and powerful.” [15]. The clustered epidemic event in Harbin province revealed that the family neighborhood is the first front of epidemic prevention, and a harmonious and stable family relationship is undoubtedly the prerequisite guarantee for intervening epidemic transmission. Under the influence of major public health events, although family relationship level is between 2.201~2.507 among different groups with unclear differences and the gap between various groups is not significant, there is still a difference among different groups when divided by occupation, age and education. In term of occupation type, the family interaction between farmer and retiree average values of 2.395 and 2.369, respectively, which are lower than those of other respondents. As far as gender was concerned, 35% of female respondents were terrified, which is two percentage points higher than that of males. In short, women were more likely to feel anxious and depressed than men and prone to conflict and tension with their families, and this is basically consistent with the research results of Cheng and Qing [18,34]. Additionally, respondents with a better neighborhood relationship whose family relationship significantly become better account for 27.8% of respondents, as compared with only 22.7% of respondents’ family relation improving when in a worse neighborhood relationship. To some extent, the possibility of the family relationship becoming better for respondents with a better neighborhood relationship is much higher than that of respondents with a worse neighborhood relationship [38,39]. With regard to age, the respondents over 60 feeling anxious account for 37.6%, while only 29.8% of those under thirty feel depressed, probably because older people are more likely to be infected than young people. There is no doubt that the family and the neighborhood are the most extensive and important front of epidemic prevention, but are also the most easily ignored. Harmonious and stable family relations are undoubtedly the basis of establishing a victory over the epidemic. It is necessary to establish the policy of “a community of shared destiny” and “a community of shared responsibility” for family and neighborhood to promote family relations and stabilize the community foundation. Therefore, the government’s grassroots community management should pay more attention to the psychological state and family relations of these special groups, actively guide them to participate in family affairs and have more emotional communication with their family, and pay attention to community construction to improve residents’ sense of belonging as well as enhance neighborhoods. Besides, the local government should prevent disharmony phenomena such as domestic violence and disallow medical staff to return home because of the epidemic fear. In addition, level of education has a positive influence on the change in family relations, and improvement of the public’s civilization quality and psychological quality cannot be achieved without the support of education. Therefore, the local governments should strengthen education at the family, school and community levels to improve the people’s spiritual culture, moral belief and life pursuit.

## 5. Conclusions

According to the questionnaire statistics, the interaction of family members was deepened under the influence of the new crown pneumonia epidemic (COVID-19) and the family relationship of most interviewees is generally improved, showing double peaks in rural and urban centers. Overall, the family relations of Chinese residents present a low–central and border–high spatial distribution pattern, which is consistent with the spread situation of COVID-19 in China during January and February. Meanwhile, the COVID-19 situation spread with a high–central and border–low characteristic. In short, the epidemic situation makes people think about the meaning of life and cherish what they have. That is to say, the more serious the epidemic situation for a province, the more family neighborhood relations improved in that province. It is necessary to establish the policy of “a community of shared destiny” and “a community of shared responsibility” for family and neighborhood to promote family relations and to stabilize the community foundation in order to solidify the premise of achieving victory over the epidemic.

In addition, according to the results of the survey, the mean value of family relationship interaction among different groups ranged from 2.201 to 2.507. The overall communication and interaction among family members generally increased, but the change was not drastic and the gap among different groups was not significant, which essentially amounted to no difference. This shows that the impact of public health emergencies on all families is the same, and since the impact of public health events is sudden, the time node is very strong and the changes in family relationships caused by this may also be abrupt, rather than the subtle influence of family members observed over many years. Regardless of the individual’s occupation, age, education level and family scale, the most important thing for family relationships is the time spent together, and the companionship of the family members is fundamental to family harmony and stability. In particular, the government and community management should pay more attention to women, older persons, single parent families and small nuclear families, and those who were more likely to feel anxious and depressed, ‘and even more prone to being infected. Through actively guiding them to participate in family affairs, and having more emotional communication with their family and neighbors.

The shortcoming of the study is the nonrandom sampling based on the network invitation and the fact that the sample is not representative of the whole population. For example, there is a lack of information about lower education groups, farmers, retirees and other professionals, while the number of highly educated people, enterprises and institutions employees and students is on the high side, meaning that extrapolation of the conclusion is limited to a certain extent. Nevertheless, we believe that our findings provide meaningful results with useful implications for policy makers and provide a reference for social study. This is probably because during that time, the whole country was kept in rigorous quarantine, meaning that people could only stay at home, which rendered a face-to-face interview impossible. In the future, more detailed work will be conducted, a questionnaire survey will be collected online and offline at the same time, and a wider range of people will be covered.

## Figures and Tables

**Table 1:** Variable setting and meaning in the model.

Variables	Variable-Definition
*status*	Family relationship level (1 = increased significantly, 2 = slightly increased, 3 = no change, 4 = slightly decreased, 5 = decreased significantly)
*edu*	Level of education (1 = primary school and below, 2 = junior high school, 3 = high school or secondary school, 4 = college and above)
*gender*	Gender (1 = male, 0 = female)
*scale*	Family size (number of households living during the outbreak)
*occupation*	Occupational types (1 = employees of enterprises and institutions, 2 = middle level and above leading cadres, 3 = entrepreneurs, 4 = students, 5 = peasant, 6 = retiree, 7 = others)
*mood*	Are you anxious or depressed about the severe form of the epidemic? (1 = yes, 0 = no)
*neighborhood*	Neighborhood relations (1 = significantly better, 2 = better, 3 = unchanged, 4 = worse, 5 = significantly worse)

**Table 2:** The average family interaction of different groups.

Family Scale	Average Family Interaction	Occupation Type	Average Family Interaction	Age	Average Family Interaction	Education Level	Average Family Interaction
2	2.466	government employee	2.270	<20	2.329	primary and below	2.507
3	2.288	middle-level cadres	2.271	20–29	2.252	junior middle school	2.383
4	2.244	entrepreneur	2.281	30–39	2.251	high schools	2.391
5	2.217	student	2.271	40–49	2.306	college and above	2.267
6	2.213	peasant	2.395	50–59	2.364		
7	2.201	retiree	2.369	>60	2.482		
		other	2.347				

**Table 3:** Correlation between family relationship and influencing factors.

Item	Age	Gender	Education Level	Occupation Type	Family Scale	Neighborhood Relations	Psychological State
Pearson Correlation	0.013	−0.058 **	−0.040 **	0.018 **	−0.080 **	0.039 **	0.013 *
Double tail significance	0.051	0.000	0.000	0.006	0.000	0.000	0.037

Note:* and ** indicate significant at 10% and 5%, respectively.

**Table 4:** Simulation results of interaction among family members.

Item	Regression Coefficient	Clustering Robust Standard Error	Wald Test Value	95% Trust Interval	
				Lower Limit	Superior Limit
occupation = governmental employee	−0.176 ***	0.050	12.369	−0.273	−0.078
occupation = middle-level cadres	−0.237 ***	0.066	12.734	−0.367	−0.107
occupation = entrepreneur	−0.258 ***	0.084	9.323	−0.423	−0.092
occupation = student	0.017 ***	0.048	0.134	−0.076	0.111
occupation = peasant	−0.049	0.107	0.214	−0.258	0.160
occupation = retiree	−0.140	0.122	1.303	−0.379	0.100
gender	0.240 ***	0.025	93.471	0.191	0.288
psychological state	−0.098 ***	0.025	15.023	−0.148	−0.049
family scale = 1	1.110 ***	0.092	144.236	0.928	1.291
family scale = 2	0.568 ***	0.065	75.943	0.440	0.695
family scale = 3	0.195 ***	0.055	12.696	0.088	0.302
family scale = 4	0.100 *	0.055	3.282	−0.008	0.208
family scale = 5	0.033	0.059	0.309	−0.082	0.147
family scale = 6	0.040	0.066	0.372	−0.089	0.169
education level = primary and below	0.441 ***	0.163	7.361	0.123	0.760
education level = junior middle school	0.247 ***	0.080	9.591	0.091	0.404
education level = senior secondary school	0.244 ***	0.050	23.668	0.145	0.342
neighborhood relations = significant better	−0.640 ***	0.137	21.837	−0.908	−0.372
neighborhood relations = slightly better	−0.584 ***	0.128	20.745	−0.836	−0.333
neighborhood relations = no change	−0.354 ***	0.119	8.854	−0.587	−0.121
neighborhood relations = slightly worse	−0.213	0.143	2.209	−0.493	0.068

Note:* and *** indicate significant levels of 10% and 1%, respectively.

## Data Availability

The data used to support the findings of this study are available from the corresponding author upon reasonable request.
